# Impact of a performance monitoring intervention on the timeliness of Hepatitis B birth dose vaccination in the Gambia: a controlled interrupted time series analysis

**DOI:** 10.1186/s12889-023-15499-w

**Published:** 2023-03-27

**Authors:** Alieu Sowe, Fredinah Namatovu, Bai Cham, Per E. Gustafsson

**Affiliations:** 1 Department of Epidemiology and Global Health, Umeå University, Umeå, Sweden; 2 Expanded Program On Immunization, Ministry of Health, Banjul, The Gambia; 3 Medical Research Council Unit The Gambia at the London, School of Hygiene and Tropical Medicine, Bakau, The Gambia; 4 School of Public Health, Georgia State University, Atlanta, GA USA

**Keywords:** Birth dose, Timeliness, Intervention, Vaccination, Hepatitis B

## Abstract

**Introduction:**

The Hepatitis B virus that can cause liver cancer is highly prevalent in the Gambia, with one in ten babies at risk of infection from their mothers. Timely hepatitis B birth dose administration to protect babies is very low in The Gambia. Our study assessed whether 1) a timeliness monitoring intervention resulted in hepatitis B birth dose timeliness improvements overall, and 2) the intervention impacted differentially among health facilities with different pre-intervention performances.

**Methods:**

We used a controlled interrupted time series design including 16 intervention health facilities and 13 matched controls monitored from February 2019 to December 2020. The intervention comprised a monthly hepatitis B timeliness performance indicator sent to health workers via SMS and subsequent performance plotting on a chart. Analysis was done on the total sample and stratified by pre-intervention performance trend.

**Results:**

Overall, birth dose timeliness improved in the intervention compared to control health facilities. This intervention impact was, however, dependent on pre-intervention health facility performance, with large impact among poorly performing facilities, and with uncertain moderate and weak impacts among moderately and strongly performing facilities, respectively.

**Conclusion:**

The implementation of a novel hepatitis B vaccination timeliness monitoring system in health facilities led to overall improvements in both immediate timeliness rate and trend, and was especially helpful in poorly performing health facilities. These findings highlight the overall effectiveness of the intervention in a low-income setting, and also its usefulness to aid facilities in greatest need of improvement.

**Supplementary Information:**

The online version contains supplementary material available at 10.1186/s12889-023-15499-w.

## Introduction

There are an estimated 1.5 million annual new hepatitis B infections and 820,000 deaths due to hepatitis B infection [1], despite a safe and effective vaccine being available for use in routine immunization programs since the early 1980s [2, 3]. Timely hepatitis B birth dose vaccination, i.e. administered within 24 hours of birth, is critical in protecting newborns as the risk of mother-to-child transmission of hepatitis B at birth can be as high as 90% [4], and most infections occurring in early life result in chronic infections [5]. Timely vaccination is therefore recommended by WHO for introduction in all countries’ routine vaccination programs [6–8]. However, timely administration of the birth dose remains a difficult task in many countries [9–14], with global timely birth dose vaccination about 42%, and as low as 17% in the African Region [15].

In The Gambia, almost one in ten babies is at risk of hepatitis B infection from their mothers due to high Hepatitis B seroprevalence (9.2%) among pregnant women [16]. Moreover, hepatocellular carcinoma (liver cancer) due to chronic hepatitis B virus infection is the most frequent cancer in The Gambia [17, 18]. The Gambia is among the first Sub Saharan African countries to introduce the hepatitis B birth dose in their routine immunization programs [19]. The hepatitis B vaccine is considered a traditional vaccine in The Gambia and therefore fully financed by the government. Almost all (98.9%) children 12 – 23 months in the 2019/2020 The Gambia demographic and health survey received it [20]. However, timely administration of the hepatitis B birth dose has remained a big challenge. It was estimated at 1.1 % during 2004 - 2014 [12], 7% during 2015 - 2016 [9], 18.5% for children aged 12-23 months in March 2020 [20], a large discrepancy that can be attributed to either methodological differences or an actual improvement in timeliness. Nonetheless, even a potential improvement of this size is small considering the burden of hepatitis B in The Gambia and the unmet goal of timely birth dose vaccination of all newborns.

Studies on interventions aiming to improve hepatitis B vaccination timeliness are therefore critical to provide evidence to guide policy decisions. The existence of studies on interventions to improve hepatitis B birth dose vaccination timeliness in the high-income context demonstrates the importance of the birth dose and the potential challenges in its implementation [21–24]. Published studies on interventions aiming to improve hepatitis B vaccination timeliness from low- and middle-income countries, where the burden of hepatitis B is higher and birth dose administration rates lower are few [25–27]. Moreover, it has been shown that improving vaccination in a setting where coverage is on an improvement course is much more challenging [28]. Therefore, there is a need to take pre-intervention performance into consideration when assessing the impact of interventions on vaccination.

Available studies are mainly from more resourceful settings where baseline timeliness thresholds, overall, are expected to be higher and might have better monitoring systems in place. This evidence gap illustrates the need for more research in this area, specifically evaluating interventions that are feasible to implement in low-income settings with less access to resources and developed infrastructure.

In 2017, The Gambia piloted a hybrid electronic immunization register, called MyChild Solution, through the *Every Child Counts* project [29], thereby facilitating the possibility to monitor hepatitis B vaccination within 1 day of birth. The project was piloted in four health facilities in two of the seven health regions of The Gambia in 2017. All health facilities in the two pilot regions were covered by this new vaccination data capture system in 2020 and in 2021, the system was rolled out nationally. Prior to the implementation of the project, vaccination histories were recorded in paper-based registers. One of the key features of the MyChild Solution is the ability to auto-calculate and display pre-defined indicators on a dashboard (accessible to users) and also send out SMS messages to valid phone numbers registered in the system. The national immunization program leveraged the monitoring potential of the solution and, in January 2020, introduced a hepatitis B vaccination timeliness indicator which was monitored monthly at the health facility level. Apart from the newly introduced monitoring intervention, all other national activities around hepatitis B birth dose timeliness remained the same for all health facilities in the country.

Based on the above background, there is a need to evaluate the impact of the monitoring intervention on monthly hepatitis B birth dose timeliness rates in the implementing health facilities in The Gambia. For this reason, we set out to assess whether 1) the intervention resulted in hepatitis B birth dose timeliness improvements overall, and 2) if the impact differed in health facilities with different pre-intervention performance using controlled interrupted time series analysis. A controlled interrupted time series analytical approach is the strongest quasi-experimental design for measuring the impact of population interventions that are well delineated in time because of its ability to account for possible time-varying confounding [30].

## Methods

### Setting

The Gambia has seven health regions. About 60% of the national population resides in Western Region 1 and Western Region 2 [31]. These two regions are more urban than the rest of the country, with Western Region 1 classified as a fully urban region using Gambia Bureau of Statistics benchmarks [20]. Since vaccination coverage in the Gambia is higher in rural than urban areas [32], it is unsurprising that the other regions have higher vaccination coverage than the two western regions. In 2017, the Ministry of Health (MoH) of The Gambia and some of its partners piloted a hybrid electronic immunization register, MyChild Solution, in the two regions [29]. The solution was fully implemented in all health facilities carrying out vaccination services in the 2 western regions in 2020. The first Covid-19 case in The Gambia was reported 10^th^ March 2020 and Covid-19 vaccination was launched 10^th^ March 2021.

The Expanded Program on Immunization and the Maternal and Child Health program (now Reproductive, Maternal, Neonatal, Child, and Adolescent Health program), are integrated at service delivery levels and offered by teams of health workers [33]. Two service delivery strategies are used: 1) fixed sessions usually based in health facilities and 2) regular outreach sessions to designated outreach sites. When brought for vaccination, children are registered in an immunization register and issued an infant welfare card at no cost, also called a home-based record, for recording vaccinations, growth monitoring, and health facility consultations amongst other uses. After they have been vaccinated, their vaccination information is updated in the register and home-based record. For birth doses, health workers are expected to follow the same approach. In the event that a home-based record is not readily available to record birth dose vaccinations, health workers will register the child, administer vaccines and document the doses administered and the child’s number from the register on the child’s mother’s antenatal card or on a piece of paper that is attached to the antenatal card for later transfer to a home-based record. For the hepatitis B birth dose, children can be vaccinated at the labor ward, at a fixed session, or an outreach session.

### Intervention

The intervention was a monthly hepatitis B birth dose vaccination timeliness indicator introduced as part of the routinely monitored vaccination indicators in intervention health facilities. In 2019, the MoH and the developers of the electronic register (Shifo Foundation) agreed to include a hepatitis B vaccination timeliness indicator as part of the monthly key performance indicators (KPIs) generated by the system. This indicator was defined as the proportion of hepatitis B vaccine doses administered within 1 day of birth out of the total hepatitis B doses administered in a month. It was included as one of 19 KPIs sent to health workers in intervention health facilities each month in January 2020. In this program, the system automatically sends out SMS KPIs to private numbers of health workers responsible for vaccination in intervention health facilities each month. No SMS reminders were sent to caregivers to take their children for the hepatitis B birth dose. Health workers in the two implementing regions were informed about the new metric and encouraged to improve timely administration of birth doses as has been done routinely. They were also supplied with noticeboards (made from cardboard and wooden planks) and predesigned graph sheets. Nineteen graph sheets, each representing one vaccination KPI, are pinned on the noticeboards that are attached to walls in public health offices of vaccinating health facilities. Health workers plot their performance for each KPI when they receive SMS messages at the end of the month. By plotting the KPI values, health workers can see their performance for the month and their performance trend over time. Please see supplementary material 1 for an example of a noticeboard pinned with KPIs.

### Design

**Fig. 1:**
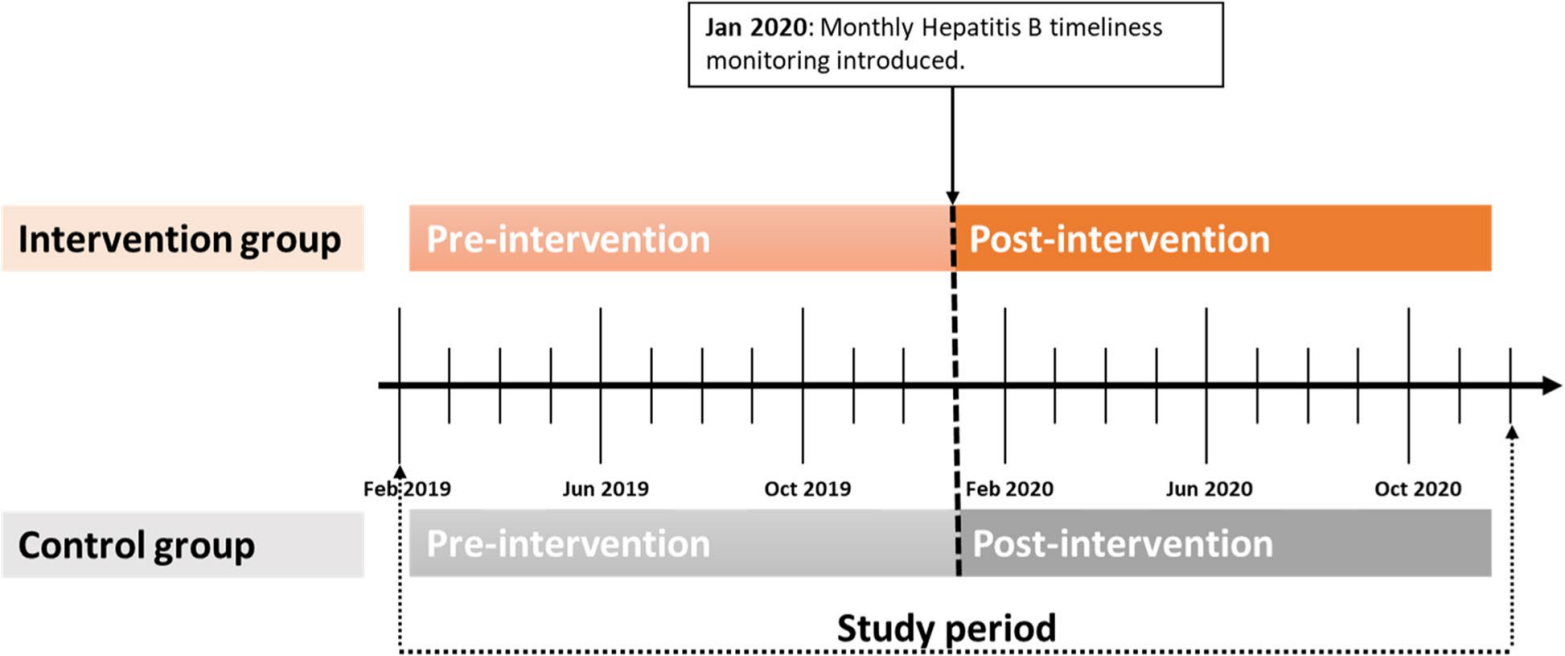
Study period showing pre- and post-intervention times for both intervention and control health facilities

We employed a controlled interrupted time series (cITS) study design to evaluate the impact of routine performance monitoring on hepatitis B vaccination timeliness (Figure 1). The pre-intervention period was February to December 2019 and the intervention period was January to December 2020. January 2019 was excluded due to data incompleteness for some health facilities in January 2019. Interrupted time series is a robust design suitable for the evaluation of population level interventions with clearly defined pre- and post-intervention time points due to its ability to account for secular trends [34–37]. Considering the potential of time-varying confounders to affect results of ITS without controls [30], we used health facilities from the five non-intervention health regions as controls. This enabled us to somehow account for random variation which is generally a problem and reflects the scientific challenges of causal inference in relation to real-world observational data.

Vaccination data for intervention health facilities (in western regions 1 & 2) was downloaded from the MyChild Solution register. Health care workers who carry out and record vaccinations and one of the researchers (AS), manually extracted data from registers in control health facilities. Steps were taken to enhance data quality before, during, and after data collection. For example, data was extracted only from health facilities with all registers for all their vaccination sites available. This is because health facilities can have several concurrent registers depending on their scheduled number of fixed and outreach vaccination sites per month. Even after health registers have been seen, data collectors screen registers for completeness of session data before starting to abstract the data.

The following information was extracted: children born and registered during the study period; having a complete date of birth; having a complete hepatitis B vaccination date; vaccinated with monovalent hepatitis B vaccine less than 60 days of birth; and a birth date not in the future of hepatitis B vaccination date. For data from the electronic register, there was no missingness in date of birth or vaccination day but 976 children were recorded as having received the hepatitis B vaccine from 60 days of birth and 938 had their date of birth in the future of their vaccination dates. Control health facilities had no missingness in date of birth and vaccination session date and no date of birth in the future of the vaccination date. However, 677 children were excluded for receiving the vaccine from 60 days of birth onwards. After all these exclusions, 41,720 and 16,972 children were analyzed for intervention and non-intervention facilities.

Monthly hepatitis B birth dose vaccination timeliness rates were calculated for both intervention and control health facilities. For each health facility, the monthly denominator included all children vaccinated with hepatitis B monovalent vaccine during that month and the numerator consisted of children vaccinated on time considering the above inclusion.

Before conducting the main analysis, we individually matched each intervention health facility with a control health facility based on their pre-intervention level and trend, in order to ensure treatment and control facilities were comparable before the intervention was implemented. Segmented regression models were fitted to compare the pre-intervention characteristics of each of the 18 intervention facilities with the 13 control health facilities. Health facilities were considered matched in cases of non-significant (*p* ≥ 0.1) pre-intervention mean level and trend differences (see statistical analysis below) [38]. Using this approach, one control health facility could be the match for one or several treatment facilities. At the end of the matching process, 16 intervention facilities were found to be comparable with 13 controls and therefore included in our analysis.

### Statistical analysis

We analyzed and presented the proportion of hepatitis B doses administered within 1 day of birth for the 16 intervention and 13 control health facilities before and after the implementation of monthly hepatitis B timeliness monitoring intervention.

Multi-group segmented regression was used to assess the impact of the intervention on hepatitis B vaccination timeliness. When one or more control groups are included, the equation of the segmented regression model can be written as follows [38].

$$Yt\;=\;\beta0\;+\;\beta1Tt\;+\;\beta2Xt\;+\;\beta3XtTt\;+\;\beta4Z\;+\;\beta5ZTt\;+\;\beta6ZXt\;+\;\beta7ZXtTt\;+\;\epsilon{t}$$

Where:

$Y_t$ = hepatitis B timeliness rate at month t;

$Z$ = dummy variable depicting treatment (intervention = 1 and control = 0);

$T_t$ = months since the study started (since February 2019 in our case);

$X_t$ = dummy variable indicating the hepatitis B timeliness intervention period (pre = 0 and post = 1) at month t and;

$ZT_t$,$ZX_t$,$ZX_t T_t$ = interaction terms created from the variables mentioned above. β0, β1, β2, and β3 represent the pre-intervention average hepatitis B timeliness rate, pre-intervention timeliness trend, post-intervention timeliness level difference, and post-intervention trend respectively in the non-intervention group. β4 is the first pre-intervention mean baseline timeliness level difference between intervention and controls while β5 captures the trend difference between the same groups over the pre-intervention period. Β6 is a measure of the first average hepatitis B timeliness difference between treatment and control groups when the intervention was introduced and β7 is the pre- and post-intervention slope difference in hepatitis B timeliness between the treatment and control groups. In addition to the 7 estimates, β0 – β7, we also estimated the post-intervention trend difference between the intervention and controls. For each estimate, we also presented its 95% confidence interval. Point estimates including a zero were considered statistically non-significant.

For the first aim, all intervention facilities were compared to all controls, and we performed segmented regression comparing monthly hepatitis B timeliness rates between the two groups.

For the second aim, we then divided the health facilities (intervention and control health facilities) into health facilities with a positive pre-trend (n: intervention = 9 and control = 10), those with a negative pre-trend (n: intervention = 5 and control = 6), and those with a flat pre-trend (n: intervention = 2 and control = 2) to assess whether the intervention differentially impacted these groups of health facilities. It should be noted that some of control health facilities matched with more than 1 intervention health facility as indicated above. For a treatment facility to be classified as having a positive pre-trend, it needed to have a p < 0.1 positive pre-tend. We used the same approach for the negative pre-trend group except that the trend had to be negative. Health facilities with a *p* ≥ 0.1 pre-trend were categorized as flat. Segmented regression was then carried out for each of the three pre-trend groups and their controls.

We considered a lag period of one month following implementation in all the regression models assuming the effect of the intervention will start after health workers have received the first SMS messages indicating their hepatitis B timeliness rates for the previous month. We tested for autocorrelation up to eleven lags and it was present at lags three and four. We fitted models with three and four lags and found their results to be comparable with the one lag model suggesting that the one lag model reasonably accounts for autocorrelation. Though the confidence intervals changed in some instances by decimal points, the point estimates were exactly the same and whether a point estimate is significant or not is the same in all the three models compared.

To provide a more comprehensive picture of the potential effect of rural/urban location of health facilities, we additionally analyzed monthly hepatitis B birth dose vaccination timeliness rates for intervention and control health facilities after the intervention was rolled out to the control group of facilities (supplemental material 2). This analysis covered the period January 2021 to April 2022, just before the main cadre carrying out vaccination services in The Gambia embarked on industrial action. The results of the analysis suggest that the location of health facilities did not impact our results. Stata version 17 was used to perform all statistical analysis [39].

## Results

The total numbers of children included in the intervention and non-intervention areas were 41,720 and 16,972 respectively. Of the 41,720 from the intervention regions, 19,783 were vaccinated in the pre-intervention period whilst the remaining 21,937 were vaccinated post-intervention. Of the 16,972 children from the control health facilities, 9,075 were vaccinated in 2020. The exclusion of January 2019 contributed to the lower total number of children in the pre-intervention era observed.

**Fig. 2:**
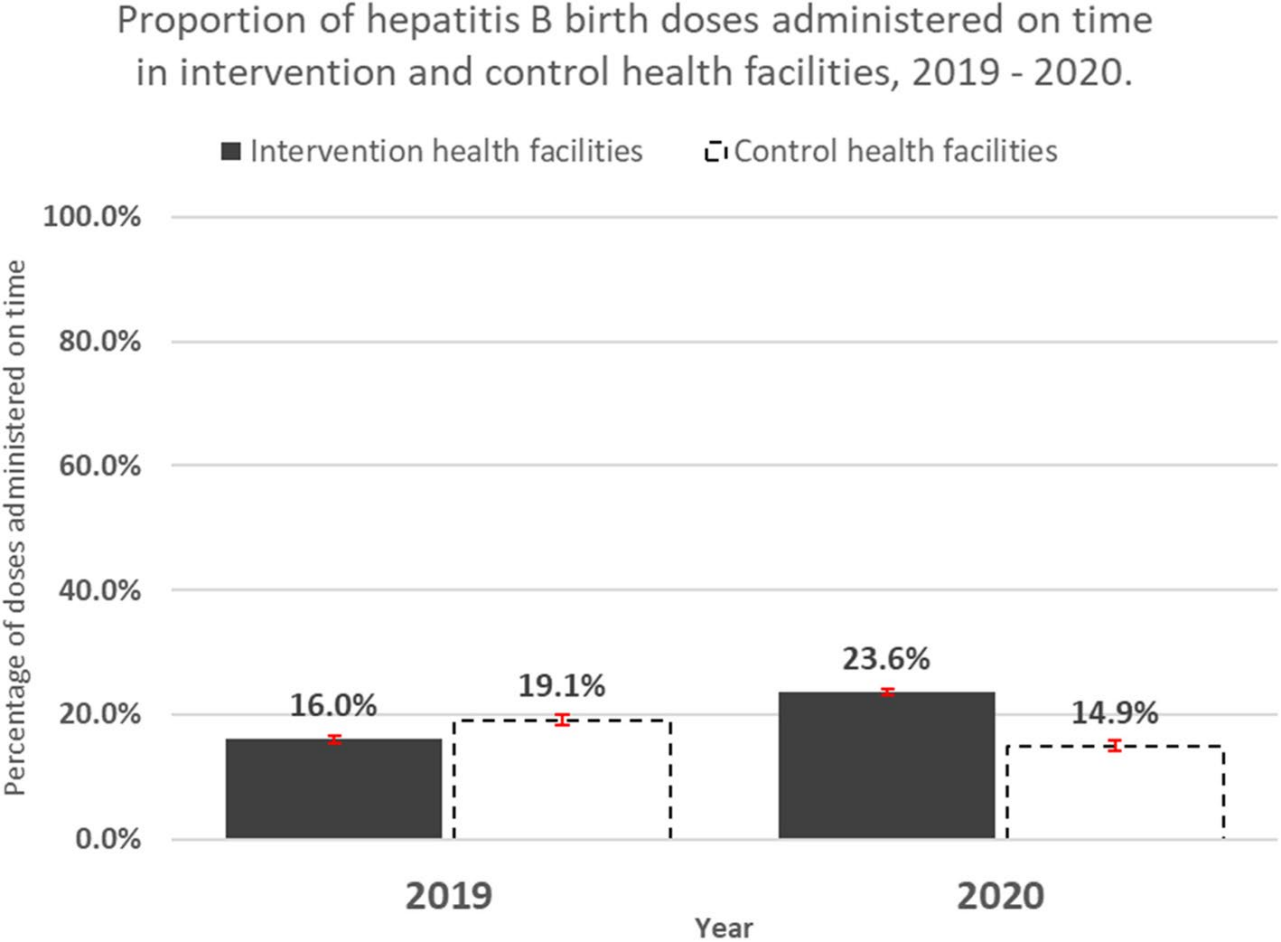
A comparison of mean hepatitis B vaccination timeliness in intervention and control health facilities, 2019—2020

Figure 2 below suggests an increase in timely administration of Hepatitis B vaccine in intervention health facilities during the period 2019 – 2020 and a decrease in control facilities during the same period. The average timeliness rate was lower in the intervention group in 2019 but higher in the same group in 2020 than the control group.

**Fig. 3:**
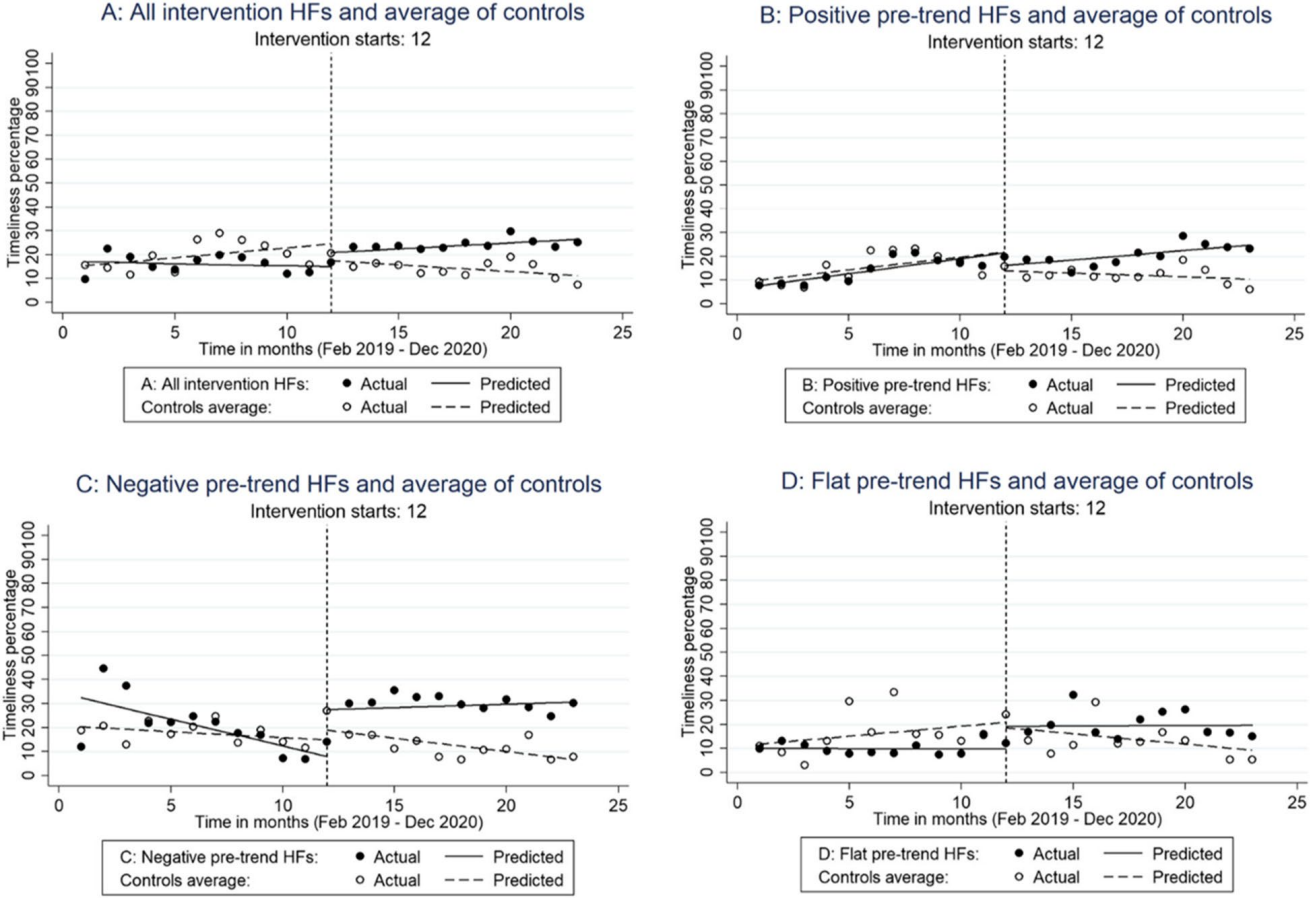
Graphs of hepatitis B vaccination timelines intervention and control health facilities, February 2019 – December 2020, The Gambia

The intervention and control groups were reasonably balanced at baseline based on their non-significant pre-level and pre-trend differences, as shown in Table 1 and illustrated in Fig. 2. Overall, considering all health centers for the first aim, mean hepatitis B vaccination timeliness improved in the intervention group post intervention to a greater degree than in the control group, as depicted by the positive post-level difference between intervention and control health facilities. The post-trend suggests that not only did the mean timeliness improve within the intervention group after the introduction of timeliness monitoring, but it also showed an increasing trend in performance. The intervention impact, estimated by the pre-post trend difference, indicated a 2.1% higher rate of timeliness improvement in the intervention group following the intervention, relative to the pre-intervention trend and corresponding trends in the control group. As shown in the graph on panel A of Fig. 3 below, mean timeliness and trend worsened in the control group in 2020 compared with 2019.

**Table 1:** Segmented regression analysis of hepatitis B vaccination timeliness comparing intervention and control health facilities in The Gambia, 2019 – 2020

**Measure**	**All intervention health facilities (HFs) (*n* = 41,720)**	**Intervention HFs with increasing pre-treatment vaccination timeliness (*n* = 20,596) **Percentage (95% CI)	**Intervention HFs with decreasing pre-treatment vaccination timeliness (*n* = 15,809) **Percentage (95% CI)	**Intervention HFs with plateaued pre-treatment vaccination timeliness (*n* = 5,315)** Percentage (95% CI)
Pre-mean level (control health facilities)	**15.39% (10.85% to 19.93%)**	**10.07% (4.94% to 15.2%)**	**20.33% (17.28% to 23.39%)**	**11.84% (2.94% to 20.73%)**
Pre-trend (control health facilities)	0.82% (-0.23% to 1.87%)	1.06% (-0.08% to 2.21%)	-0.5% (-1.06% to 0.06%)	0.83% (-0.49% to 2.14%)
Post-level difference (control health facilities)	-6.99% (-16.93% to 2.96%)	-7.85% (-17.84% to 2.13%)	4.06% (-4.13% to 12.26%)	-2.24% (-15.15% to 10.66%)
Post-trend (health facility)	**-1.39% (-2.51% to -0.27%)**	**-1.38% (-2.64% to -0.12%)**	-0.61% (-1.45% to 0.23%)	**-1.68% (-3.31% to -0.04%)**
Pre-level difference (intervention – control health facilities)	1.6% (-5.43% to 8.64%)	-2.40% (-8.03% to 3.24%)	12.04% (-3.16% to 27.24%)	-1.83% (-11.22% to 7.57%)
Pre-trend difference (intervention – control health facilities)	-1.01% (-2.37% to 0.35%)	0.19% (-1.11% to 1.49%)	-1.72% (-3.92% to 0.48%)	-0.83% (-2.3% to 0.63%)
Post- level difference (intervention – control health facilities)	**12.92% (1.57% to 24.28%)**	2.5% (-10.03% to 15.02%)	**15.47% (0.48% to 30.47%)**	11.53% (-4.89% to 27.96%)
Post-trend difference (intervention – control health facilities)	**1.07% (0.31% to 1.83%)**	**1.09% (0.23% to 1.96%)**	1.39% (-0.11% to 2.88%)	0.90% (-0.62% to 2.41%)
Pre- post-trend difference (intervention – control health facilities)	**2.08% (0.56% to 3.61%)**	0.90% (-0.57% to 2.38%)	**3.11% (0.42% to 5.80%)**	1.73% (-0.25% to 3.70%)

**Note:** In the table above “pre” refers to pre-intervention period and “post” refers to post intervention period

Corresponding to the second aim, stratifying the health facilities indicated that the intervention impact differed by pre-intervention performance (timeliness trend and levels). First, in the group of health facilities with a positive pre-trend, both treatment and controls seemed to experience immediate drops (Figure 3: panel B) in monthly average timeliness rates post intervention. While the control health facilities continued to have subsequent lower monthly timeliness rates [post-trend health facilities = -1.38% (-2.64% to -0.12%)], facilities in the intervention group picked up and continued improving thereupon [intervention – control health facilities post-trend difference = 1.09% (0.23% to 1.96%)]. This resulted in a non-significant and small intervention impact [pre-post-trend difference = 0.90% (-0.57% to 2.38%)]. Second, the health centers in the group with decreasing performance over the months prior to the intervention made a substantial gain [15.47% (0.48% to 30.47%)] in mean timeliness when the intervention was implemented. The group continued along an improvement path as can be deduced from panel C of Figure 3 and the pre-post trend difference in Table 1. This was expressed in a significant intervention impact of greater magnitude than in the total sample [pre-post-trend difference = 3.11% (0.42% to 5.80%)]. Third, health centers classified as having a plateaued performance pre-intervention made no statistically significant gains on hepatitis B vaccination timeliness in either level or trend though this seemed to improve visually (panel D of figure 3). The control health facilities of this group (flat trend) manifested a declining performance after the introduction of the performance monitoring intervention. The resulting intervention impact was non-significant, albeit of a comparable magnitude as in the total sample [pre-post-trend difference = 1.73% (-0.25% to 3.70%)].

## Discussion

Our aim was to evaluate the impact of a monthly hepatitis B vaccination timeliness monitoring intervention on timely hepatitis B vaccination rates and whether the intervention impact was differential by health facility performance prior to the intervention in The Gambia. Our study showed that, overall, timeliness improved in the intervention health facilities than control health facilities taking pre-intervention trends into account. This overall intervention impact was, however, dependent on pre-intervention health facility performance, with large impact among poorly performing facilities, and with uncertain moderate and weak impacts among moderately and strongly performing facilities, respectively. The impact of the monitoring intervention is positive especially in a setting with low coverage where every improvement counts. Nonetheless, the relatively small overall improvement observed demonstrates the challenge of reaching the desired timeliness goal. The barriers of hepatitis B birth dose administration are complex and composed of a myriad of factors at multiple levels e.g. policy, health facility and community levels [19]. This complexity illustrates the need to implement the WHO triple elimination strategies for HIV, viral hepatitis, and syphilis as an opportunity to improve timely hepatitis B birth dose administration [40].

Hepatitis B vaccination at birth is effective in protecting against hepatitis B virus infection and presents an important component to achieve global elimination of the disease [3, 8, 41, 42]. Our study is among the first to examine the impact of a population level intervention to increase hepatitis B birth dose timeliness in a low- and middle-income country setting. In comparison with studies from the US, we evaluated the impact of introducing a monitoring mechanism in a low-resource setting with low timeliness rates, and those in the US evaluated other interventions added to an already existing monitoring system in a setting with relatively higher timeliness rates. Though the interventions and settings are different, from a general perspective, our finding that the intervention generally increased timely administration of the hepatitis B birth dose is similar to what the US studies reported [22–24]. Similarly, studies in Asia found interventions they evaluated to have led to improvements in time administration of the hepatitis B birth dose [25–27]. The positive gains reported by hepatitis B timeliness intervention studies signal signs of hope that despite the obvious challenges towards achieving optimal birth dose coverage, it can be achieved.

Our finding of immediate and sustained overall improvement in timeliness rates post-monitoring implementation suggests that an intervention as simple as visualizing performance to service providers could trigger improvement actions. Nonetheless, the intervention could have heterogenous effects on population groups with contrasting pre-intervention performance trends as observed in our study. It is thought-provoking that the group of facilities on an improvement course made no significant immediate timeliness gains after the intervention though they made noticeable gains upwards in comparison with their control health facilities. The fact that this group had a lower pre-intervention intercept and was already on an upward trajectory could contribute to explaining this phenomenon. Using the same line of reasoning, the significant improvements in both level and trend post-intervention in the group of negative pre-trend (though they had the highest pre-intervention timeliness level) facilities observed becomes expected. The finding that the intervention was most helpful in poorly performing health facilities matching previous research [28] is a positive one, because this is the group where improvement is needed the most.

### Strengths and limitations

The main strength of our study is the use of a robust methodological design ideal for assessing population level interventions. Baseline dissimilarities in pre-intervention levels or trends between comparison groups are a challenge to validity of cITS results. We accounted for this by matching the intervention and control groups on their levels and trends before the implementation of the monitoring intervention. Based on the statistically insignificant difference between sets of intervention health facilities and their comparators in pre-intervention timeliness rates and trends, we can assume that the intervention and control groups in our study were sufficiently comparable for yielding valid estimates.

The use of 11 pre- and 12 post-intervention monthly data points may be sufficient to estimate the immediate and over the course of time impacts of the intervention [35]. Monthly aggregate data may appear short for evaluating the effect of an intervention. However, routine vaccination coverage and its related indicators are normally measured and monitored monthly. Also, we hypothesized that the intervention would have an immediate impact meaning that health workers will be triggered to react immediately. This immediate reaction to improving timeliness rates will be measurable in the subsequent month. Notwithstanding, we acknowledge that a post-intervention period of one year may not provide adequate insight on impact of the timeliness monitoring intervention in the long term.

The intervention area is more urban than the rest of the country, contains more health facilities (where vaccines are kept), and also tends to have a higher proportion of facility-based deliveries [20]. This could potentially introduce bias in the estimation of intervention impact, especially considering the surprising post-intervention negative trend noticeable in all the control groups. The negative trend in control health facilities may potentially be due to disruptive impact of the Covid-19 pandemic on vaccination efforts. While the pandemic hit both control and intervention facilities and as such is not a selective exposure in the control group, the intervention may have partially counteracted the impact of the pandemic in intervention health facilities specifically when it comes to hepatitis B birth dose timeliness. It is noteworthy that, on average, institutional delivery even in the rural areas is at least 75% (84% national) based on results of the 2019/2020 demographic and health survey [20]. Therefore, it appears unlikely that differences in health facility-based delivery rates are responsible for the generally poorer trend of the control health facilities observed in this study. The difference in institutional deliveries will likely come into play only after birth dose timeliness rates approach the average institutional delivery frequency of rural areas. The results of our supplementary analysis (supplemental material 2) support this point. It has been reported that being born in a health facility has no effect on hepatitis B timeliness in The Gambia [12] unlike in other settings, such as Colombia and Senegal [11, 14].

The Ministry of Health’s annual service statistics report revealed that, overall, there was a slightly higher proportion of institutional deliveries in 2020 than 2019 (96.5% vs 94.8%) [43]. Since this study included children captured in the health facility immunization registers, it is possible that some eligible children were missed, especially taking into account the impact of the Covid-19 pandemic on routine immunization [44]. Including the potentially missed children could raise or decrease timeliness rates depending on their time to hepatitis B vaccination.

Not all health facilities offering vaccination services in the country were included in our study. Therefore, it was not possible for us to determine the proportions of health facilities with negative, flat, or positive pre-intervention trends. However, other published studies on the effectiveness of interventions on hepatitis B birth dose timelines principally used sub-populations [21–27]. The immunization registers contained no information on place of delivery. So, it was not possible to take that into account. The number of health workers available for vaccination services may affect vaccination rates. This information was not collected and therefore not analyzed. The degree to which the intervention was (in) effective could also be influenced by how well it was maintained, e.g., regular updating of the charts. This information is not available and thus not considered in this evaluation of the intervention. Investigating the importance of intervention maintenance is a potential topic for future research. Another important research area is the identification of factors driving the low hepatitis B vaccination rate in The Gambia. Finally, a challenge for interventional studies especially at the population level is contamination bias, which would lead to an underestimation of the impact of the intervention. Based on the post-intervention performance of the controls, contamination bias seems unlikely, but can nevertheless not be completely ruled out.

## Conclusions

The implementation of a novel hepatitis B vaccination timeliness monitoring system in health facilities led to overall improvements in both immediate timeliness rate and trend. The intervention was especially helpful in poorly performing health facilities highlighting a differential impact on health facilities with different pre-intervention performance characteristics. This differential impact of the monitoring intervention should be considered where possible in designing future interventions for all health facilities to maximally benefit. Our results suggest that despite a positive impact of the intervention observed, on its own it is unlikely that it will be able to raise birth dose timeliness to the desired level. Therefore, the national immunization program may wish to try other interventions to complement performance monitoring considering the multifactorial nature of hepatitis B birth dose administration.

## Supplementary Information

**Additional file 1.**

**Additional file 2.**

## Data Availability

The datasets analysed during the current study are available from the corresponding author on reasonable request.
