# Supplementary material for: Impact of a performance monitoring intervention on the timeliness of Hepatitis B birth dose vaccination in the Gambia: a controlled interrupted time series analysis
Source: BMC Public Health. 2023 Mar 27;23:568. doi: 10.1186/s12889-023-15499-w (PMC10041491; doi:10.1186/s12889-023-15499-w)

Supplementary figure 1: A picture showing charts for monitoring key vaccination performance indicators attached to a noticeboard

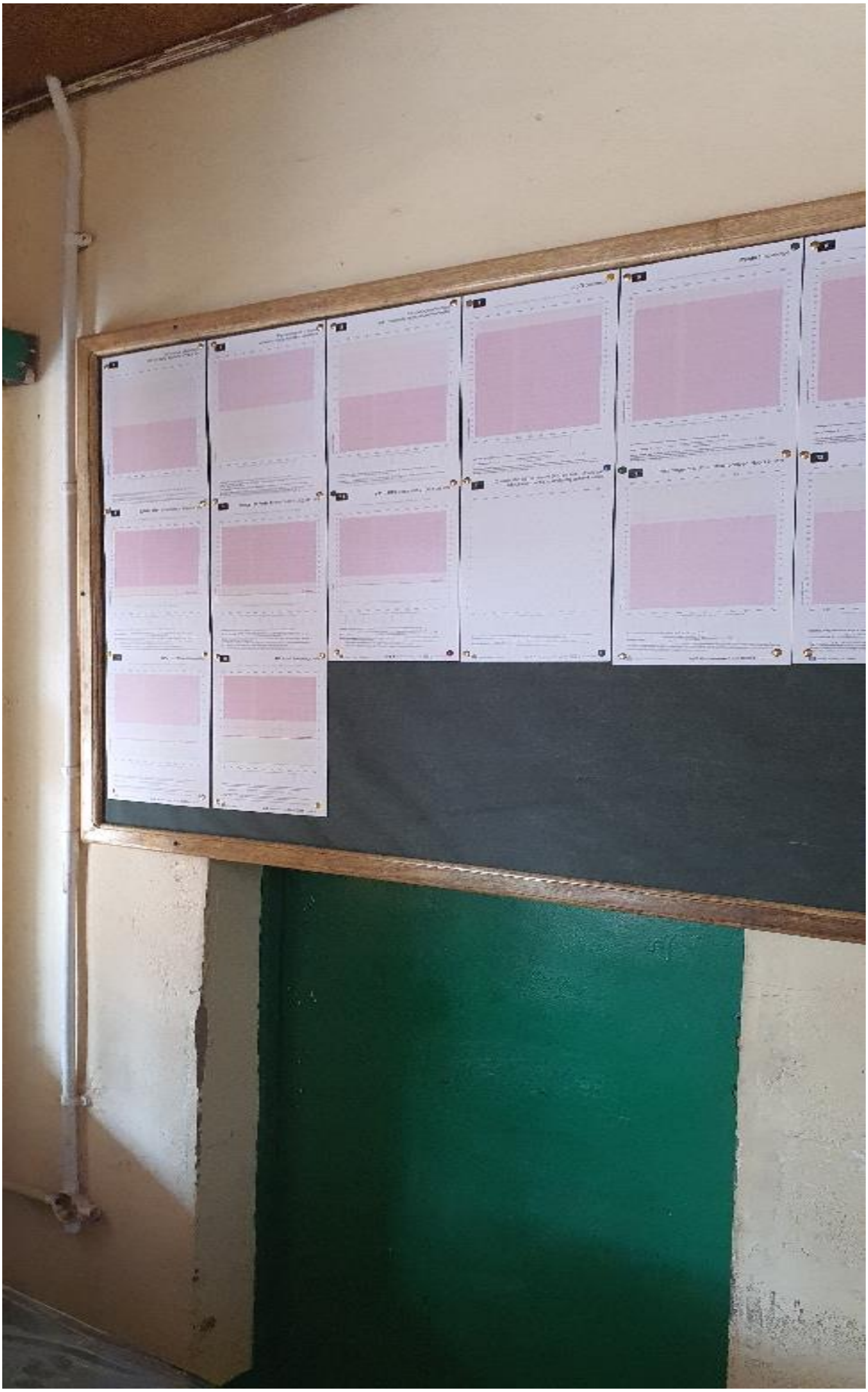

Supplement: Supplementary file 1 — Additional file 1. [file 12889_2023_15499_MOESM1_ESM.pdf]
