# Supplementary material for: Impact of a performance monitoring intervention on the timeliness of Hepatitis B birth dose vaccination in the Gambia: a controlled interrupted time series analysis
Source: BMC Public Health. 2023 Mar 27;23:568. doi: 10.1186/s12889-023-15499-w (PMC10041491; doi:10.1186/s12889-023-15499-w)
Supplement: Supplementary file 2 — Additional file 2. [file 12889_2023_15499_MOESM2_ESM.pdf]

Supplementary Table 1: Timely administration of hepatitis B birth dose in intervention and non-intervention health facilities in The Gambia, January 2021 – April 2022.

Period	Non-intervention health facilities		Intervention health facilities		Total sample	
	Total	Number and percent vaccinated on time	Total	Number and percent vaccinated on time	Total	Number and percent vaccinated on time
Year = 2021						
January	400	178 (44.5%)	1433	654 (45.6%)	1833	832 (45.4%)
February	834	180 (21.6%)	1951	690 (35.4%)	2785	870 (31.2%)
March	783	151 (19.3%)	2053	575 (28.0%)	2836	726 (25.6%)
April	727	126 (17.3%)	1737	441 (25.4%)	2464	567 (23.0%)
May	612	122 (19.9%)	1412	337 (23.9%)	2024	459 (22.7%)
June	804	189 (23.5%)	1499	432 (28.8%)	2303	621 (27.0%)
July	751	218 (29.0%)	1380	551 (39.9%)	2131	769 (36.1%)
August	686	236 (34.4%)	1263	524 (41.5%)	1949	760 (39.0%)
September	880	300 (34.1%)	2092	998 (47.7%)	2972	1298 (43.7%)
October	1168	413 (35.4%)	2865	1212 (42.3%)	4033	1625 (40.3%)
November	1112	299 (26.9%)	2642	977 (37.0%)	3754	1276 (34.0%)
December	1145	305 (26.6%)	2577	889 (34.5%)	3722	1194 (32.1%)
**Total**	**9902**	**2717 (27.4%)**	**22904**	**8280 (36.2%)**	**32806**	**10997 (33.5%)**
Year = 2022						
January	1302	556 (42.7%)	2797	1053 (37.7%)	4099	1609 (39.3%)
February	945	485 (51.3%)	2628	988 (37.6%)	3573	1473 (41.2%)
March	1036	407 (39.3%)	2367	989 (41.8%)	3403	1396 (41.0%)
April	750	217 (28.9%)	1827	632 (34.6%)	2577	849 (33.0%)
**Total**	**4033**	**1665 (41.3%)**	**9619**	**3662 (38.1%)**	**13652**	**5327 (39.0%)**
